# Advancements in biopile-based sustainable soil remediation: a decade of improvements, integrating bioremediation technologies and AI-based innovative tools

**DOI:** 10.1007/s11356-025-37002-1

**Published:** 2025-10-08

**Authors:** Mojtaba Ostovar, Sara Muñana, Alazne Galdames, Josu Berganza, Maider Orueta, José Julián Esteban, Pilar Brettes, José Luis Vilas Vilela, Leire Ruiz Rubio

**Affiliations:** 1 Macromolecular Chemistry Group (LQM), Physical Chemistry Department, Faculty of Science and Technology, University of the Basque Country (UPV/EHU), 48940 Leioa, Spain; 2 GAIKER Technology Centre, Basque Research and Technology Alliance, 48170 Gaiker, Zamudio Spain; 3 Iragaz Watin S.A., 20720 Azkoitia, Spain; 4 Departamento de Geología, Facultad de Ciencia y Tecnología, Universidad del País Vasco/Euskal Herriko Unibertsitatea (UPV/EHU), Barrio Sarriena S/N, 48940 Leioa, Spain; 5 BCMaterials, Basque Center for Materials, Applications and Nanostructures, UPV/EHU Science Park, 48940 Leioa, Spain

**Keywords:** Biopile, Carbon Sequestration, Bioremediation, Nanotechnology, Sustainability, Phytoremediation

## Abstract

Soil degradation has become a significant global issue, driven by unsustainable practices such as intensive agriculture, deforestation, and overgrazing. These activities accelerate erosion and ecosystem deterioration, necessitating effective remediation strategies to restore soil health. Bioremediation, particularly biopile technology, offers an environmentally friendly approach by leveraging microbial activity to degrade pollutants. The efficiency of biopiles depends on key factors such as moisture, temperature, nutrient availability, oxygen levels, and microbial diversity. Additionally, sustainable materials, including nanotechnology-based solutions like nanoparticles and biosurfactants, further enhance the bioremediation process, improving pollutant removal and promoting long-term soil health. This review explores these techniques and their role in sustainable remediation. While not the primary focus, artificial intelligence (AI) tools, such as VOSviewer, provide added value by analyzing research trends and optimizing bioremediation strategies.

**Graphical Abstract:**

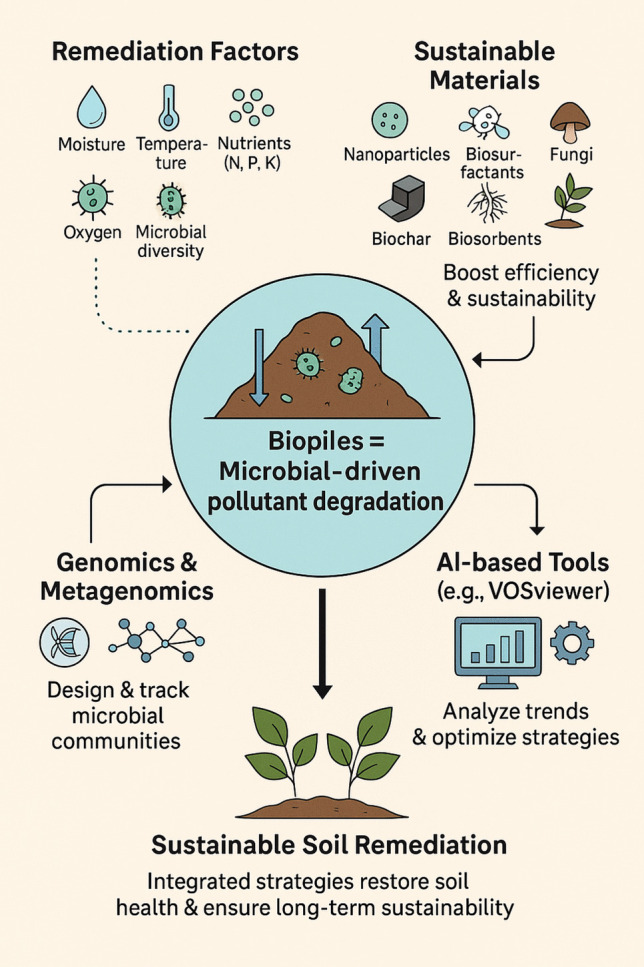

**Supplementary Information:**

The online version contains supplementary material available at 10.1007/s11356-025-37002-1.

## Introduction

In recent decades, soil degradation has become increasingly evident, being in different regions an important health and environmental problem. Environmentally unsustainable practices, such as intensive agriculture, deforestation, overgrazing, intensive cultivation, forest fires, and construction work, have accelerated soil degradation. It must be considered that soil is a limited resource; these actions deteriorate it, leaving it vulnerable to wind and water erosion. This erosion, in turn, damages the complex underlying ecosystems. In this context, the soil remediation and regeneration process plays a key role in recovering the usage and soil health.

Sustainable soil remediation is becoming increasingly important as rapid industrialization and urbanization lead to the accumulation of emerging contaminants, which threaten soil health and quality. Traditional remediation technologies often face challenges such as high costs, environmental risks, secondary pollution, and long-term contamination leaching. To address these issues, sustainable remediation approaches are essential, focusing on methods that minimize environmental impact while maximizing social and economic benefits. These strategies include using eco-friendly technologies and bioremediation techniques such as microbial bioremediation and phytoremediation, which not only remove contaminants but also restore soil health and support long-term environmental sustainability. Although bioremediation and phytoremediation are sustainable remediation methods, their sustainability and circularity could also be increased using eco-friendly materials and processes, considering these from the early design of the methodology used for soil restoration. Research into innovative, cost-effective remediation methods is crucial for advancing solutions that mitigate soil contamination while promoting sustainable land use (Lee et al. 2024).

In the case of bioremediation, which involves the use of microorganisms, it can be implemented either in situ or ex situ, depending on factors like cost, site characteristics, and the type and concentration of pollutants. Phytoremediation, another biological treatment method, utilizes plants to extract, immobilize, or degrade pollutants, contributing to the overall remediation effort. This approach is particularly beneficial in scenarios where plant-based solutions align with site conditions and treatment objectives (Azubuike et al. 2016).

This review paper provides a comprehensive overview of the last decade of biological remediation methods, with a focus on biopiles and their integration with other techniques. It assesses the principles, advantages, limitations, and potential solutions associated with microbial bioremediation, phytoremediation, and ecopiles. Emphasizing the sustainability of these bioremediation approaches, it offers a framework for their future implementation in environmentally sensitive and economically constrained contexts. This includes evaluating their effectiveness in addressing soil contamination while considering their environmental, social, and economic impacts for long-term sustainable solutions.

## Bioremediation

Bioremediation is an environmentally friendly and effective strategy for the decontamination of environments affected by toxic compounds such as heavy metals, hydrocarbons, and pesticides. This technique is based on the use of microorganisms, such as bacteria, fungi, and algae, which have the ability to degrade, transform, or immobilize pollutants, converting them into less toxic or stable substances. By metabolizing or co-metabolizing these substances, microorganisms not only decrease the toxicity of the environment but can also integrate them into their metabolic cycles as sources of carbon and energy. This approach is particularly useful for addressing persistent organic pollutants (POPs) and persistent toxic elements (PTEs), as some microorganisms can immobilize these elements in organic or inorganic complexes that reduce their solubility and mobility in soil and water (Aparicio et al. 2022; Mali et al. 2023).

### Sustainability of bioremediation techniques

Bioremediation has evolved in the last decade to become a highly effective technique to recover polluted soil. However, sustainability in bioremediation practices has become a critical focus in addressing environmental pollution, particularly as global efforts shift toward environmentally responsible and resource-efficient solutions. Among various methods, microbial bioremediation has gained attention for its ability to degrade complex pollutants with high specificity, which is briefly explained in the following sections (Bala et al. 2022). However, while this technique offers significant potential, its sustainability is influenced by a range of factors that extend beyond its immediate efficacy. Furthermore, emerging methods such as nano-bioremediation, which combines nanotechnology with microbial degradation, promise to enhance pollutant breakdown efficiency while minimizing resource consumption (Ashraf et al. 2025; Galdames et al. 2020).

#### Microbial Nanotechnology

Nanotechnology-based remediation methods, such as nano-bioremediation, indirectly enhance soil carbon sequestration by improving soil health and promoting microbial and plant activity. Engineered nanoparticles facilitate the degradation of contaminants, allowing for the restoration of soil functionality and the re-establishment of vegetation (Adnan et al. 2025). This process increases organic carbon inputs into the soil via plant root exudates and microbial activity, supporting stable carbon storage in soil aggregates. Thus, nanotechnology not only expedites remediation but also contributes to building resilient soil systems that sequester carbon effectively.

The loss of natural resources and persistent environmental challenges, such as pollution and global warming, need innovative and sustainable solutions. Microbial nanotechnology represents a promising approach in addressing these issues, merging biotechnology with nanoscience to enhance environmental remediation processes. By utilizing microbes along with nanoparticles, pollutants can be broken down more efficiently, and environmental restoration can be achieved with minimal ecological disruption (Cecchin et al. 2017; Chaudhary et al. 2023).

Microbe-based nanoparticles (NPs) are more effective and environmentally friendly compared to chemical-based NPs for pollution removal. They convert heavy metals into simpler, less harmful forms, ensuring environmental sustainability. Additionally, these NPs are cost-effective, target specific pollutants, and minimize environmental impact. Their eco-friendly nature, stability, and reduced harm to the environment make them a sustainable solution for controlling pollution (Ashraf et al. 2025; Zhang et al. 2022).

#### Sustainable Materials

Sustainable materials are revolutionizing bioremediation processes by enhancing efficiency, sustainability, and scalability in addressing environmental contamination.

Biosurfactants, biodegradable and low-toxicity amphiphilic molecules produced by microorganisms, play a crucial role in sustainable bioremediation. Their effectiveness under extreme conditions makes them ideal for environmental pollution control. Research highlights the potential of biosurfactants from renewable sources like sunflower oil in soil and water decontamination (Passos et al. 2025). These biosurfactants reduce reliance on synthetic surfactants and utilize agricultural by-products, supporting a circular economy. By lowering surface tension and enhancing the bioavailability of hydrophobic pollutants, they facilitate microbial degradation (Olasanmi and Thring 2018). Their role in waste reduction and treated effluent reuse further advances sustainable waste management (Mulligan 2021).

Patowary et al. (2018) investigated the efficacy of a biosurfactant produced by *Pseudomonas aeruginosa* SR17 in the bioremediation of oil-contaminated soils. The application of rhamnolipid biosurfactant at 1.5 g L⁻^1^ resulted in 86.1% and 80.5% degradation of total petroleum hydrocarbons (TPHs) in soils containing 6800 ppm and 8500 ppm (parts per million) TPH, respectively, which surpasses the 70.8% and 68.1% degradation achieved by sodium dodecyl sulfate (SDS). The analysis revealed the complete removal of fluoranthene, benz(b)fluorene, and benz(d)anthracene polycyclic aromatic hydrocarbons (PAHs), emphasizing the biosurfactant’s potential for enhancing biodegradation (Patowary et al. 2018).

Natural sorbents such as zeolite, kaolinite, diatomite, and vermiculite provide cost-effective solutions for reducing soil toxicity, improving water retention, and supporting microbial activity (Vasilyeva et al. 2022). These materials not only mitigate contamination but also improve soil structure, enhance plant growth, and promote long-term ecological balance, offering a sustainable pathway for environmental restoration (Srivastava et al. 2025).

Biochar, a sustainable product derived from agricultural waste through optimized pyrolysis, offers high adsorption capacity due to its large surface area, improved porosity, and adjustable surface functionality, making it effective for sequestering heavy metals and organic pollutants (Dike et al. 2021; Kong et al. 2018; Zahed et al. 2021). The application of biochar offers advantages in both carbon sequestration and soil conservation. In addition to its ability to mitigate pollutants, biochar contributes to climate change mitigation through long-term carbon storage and by reducing greenhouse gas emissions, such as N₂O and CH₄. By managing crop residues and preventing their burning, biochar not only improves soil quality but also enhances nutrient and water use efficiency, further promoting sustainability (Gupta et al. 2020; Shar et al. 2025).

The implementation of the *Trichoderma reesei*-laccase-biochar coupling system (TLBS) demonstrated remarkable efficiency in the bioremediation of heavy metals (Ni and Cd) and organic pollutants. The TLBS achieved a 93.63% reduction in Ni and 89.68% reduction in Cd bioavailability while simultaneously remediating a range of organic contaminants (71.41–96.79%), including antibiotics and pesticides (Xia et al. 2025). These results underscore the potential of TLBS as a sustainable and effective approach to addressing complex environmental contamination, leveraging eco-friendly, biodegradable materials to promote long-term environmental health and agricultural productivity.

In 2024, Dinakarkumar et al. (2024) explored the potential of fungal bioremediation as a sustainable solution to environmental pollution. They highlighted fungi’s enzymatic arsenal, including laccases, peroxidases, and hydrolases, which effectively degrade persistent organic pollutants like PAHs, PCBs, and petroleum hydrocarbons, while also immobilizing heavy metals through biosorption and bioaccumulation (Dinakarkumar et al. 2024). The versatility of fungi across soil, water, and air remediation was emphasized, along with their ability to detoxify xenobiotics.

### Biostimulation

Biostimulation is an effective and versatile technique to accelerate the biodegradation of hydrocarbons and other organic pollutants in contaminated soils. This method involves modifying the environment to enhance the activity of indigenous microbial communities capable of decomposing contaminants. Factors such as nutrient availability, pH, temperature, oxygen content, moisture, and soil structure are critical to the success of biostimulation (Hazen 2010; Omokhagbor Adams et al. 2020). The addition of essential nutrients, such as nitrogen, phosphorus, and additional carbon sources, as well as electron acceptors such as oxygen, helps to overcome the limitations of microbial biodegradation in contaminated soils (Omokhagbor Adams et al. 2020).

Recent studies have shown that the use of sustainable amendments could contribute to enhancing biostimulation while ensuring minimal environmental impact. Organic materials, such as agricultural waste, food processing by-products, and water hyacinth compost, have gained attention for their nutrient-rich properties that stimulate microbial growth.

Udume et al. (2023) found that water hyacinth (WH) and spent mushroom compost (SMC) significantly enhanced petroleum hydrocarbon degradation. The highest TPH biodegradation (93%) occurred in sterilized soil reintroduced with microbes and optimized WH compost, followed by 89% in soil treated with (SMC + WH). Inorganic NPK fertilizer achieved 86% degradation, while natural attenuation resulted in only 4%. This indicates that in the absence of nutrient-boosting amendments, natural processes alone are very slow in breaking down TPH (Udume et al. 2023).

Although biostimulation offers several advantages, including its reliance on native microorganisms and cost-effectiveness, it also faces challenges related to site-specific conditions such as soil type, pollutant composition, existing microbial communities, and environmental factors (Azuazu et al. 2023). Additionally, time constraints can lead to prolonged remediation periods, limiting its application on a large scale (Dehnavi and Ebrahimipour 2022). Another key challenge is the need for careful nutrient management, as improper nutrient balance can significantly affect soil microbial activity and the overall efficiency of the bioremediation process (Karim et al. 2024).

### Bioaugmentation

Bioaugmentation is a bioremediation strategy that involves the introduction of external microorganisms to enhance the biodegradation processes of contaminants in soil. These microorganisms can be specialized exogenous cultures, non-indigenous microbial communities, or previously isolated indigenous species with outstanding degradation capabilities. It also includes the use of genetically modified microorganisms that have been engineered or adapted to metabolize specific pollutant compounds (Chukwunonso Ossai et al. 2022). This process not only aims at removing contaminants, but also at restoring the biological functions of the soil by enhancing native microbial activity and promoting beneficial interactions between introduced and indigenous microorganisms (Aparicio et al. 2022, 2018). These interactions are essential to ensure the efficiency of the bioremediation process, as introduced microorganisms must survive in new environments, compete for resources, and move within the soil structure (Zhang et al. 2023).

Autochthonous fungal bioaugmentation (AFB) offers a sustainable approach to the remediation of petroleum hydrocarbon–contaminated soils, as demonstrated by Huang et al. (2025). In their study, *Fusarium solani* LJD-11 and *Aspergillus fumigatus* LJD-29 significantly degraded recalcitrant pollutants such as *n*-hexadecane (*n*-Hex), benzo[a]pyrene (BaP), and dibenzothiophene (DBT). Specifically, *F. solani* achieved 92.6% BaP, 87.1% *n*-Hex, and 64.3% DBT degradation, while *A. fumigatus* removed 93.7% BaP, 79.7% *n*-Hex, and 63.8% DBT. These findings show the potential of fungal bioaugmentation in breaking down persistent organic pollutants and improving soil restoration efforts. AFB not only improves pollutant removal but also supports the restoration of soil microbial diversity, which is crucial for long-term soil health (Huang et al. 2025).

Silva et al. (2015) successfully applied the bacterium *Arthrobacter aurescens* TC1 at concentrations of 5 × 10⁷ and 2 × 10⁸ cfu/g of soil to remove terbuthylazine at a concentration of 3.8 mg/kg from contaminated soils. In freshly spiked soils, bioaugmentation led to a rapid removal of 95% of pesticide within just three days, compared to only 30% removal in non-bioaugmented soils over a 14-day period (Cao et al. 2022; Goswami et al. 2018; Liu et al. 2024; Silva et al. 2015).

Liu et al. (2024) investigated its effects on soil contaminated with benzene, toluene, and trichloroethylene (TCE), demonstrating that introducing specialized microbial cultures increased pollutant removal rates from 33.02 to 37.55%. The microbial consortium, primarily composed of *Pseudomonas*,* Stenotrophomonas*, and *Chryseobacterium*, played a key role in accelerating biodegradation. Moreover, when bioaugmentation was combined with biostimulation, the overall degradation efficiency further increased to 62.38–68.84%, showing the synergistic benefits of both approaches. This paper indicates that bioaugmentation, particularly when integrated with biostimulation, is a highly effective strategy for remediating co-contaminated soils.

### Landfarming

Landfarming is considered a cost-effective and adaptable bioremediation method, generally categorized as ex situ but occasionally applied in situ based on contamination levels. This approach includes the transportation of contaminated soil to a designated area, usually close to the contaminated site, where it is spread on a bed and regularly plowed to enhance aeration, facilitating the decomposition of pollutants. Landfarming could be considered a sustainable soil remediation technology that uses microorganisms to mitigate contamination, recycle nutrients, and maintain soil properties for plant growth. Strategies like biostimulation, through nitrogen and phosphorus addition, and bioaugmentation have proven to accelerate remediation, supporting circular economy principles and enhancing sustainable soil restoration (Di Marcantonio et al. 2023; Sims and Sims 2003). In this method, the soil is excavated and placed on a treatment area, usually equipped with appropriate thin-layer spreading on the ground, or impermeable liners are employed to minimize leaching and prevent groundwater contamination (Tampouris et al. 2001).

Aeration, nutrient addition, and moisture control are key elements stimulating microbial activity, contributing to the cost-effectiveness of the process and ensuring minimal environmental impact (Maila and Cloete 2004). Frutos et al. (2012) reported that slurry bioremediation and landfarming treatments of sludge samples with a total petroleum hydrocarbon (TPH) content of 2243 mg/kg yielded a TPH reduction efficiency of 57% and 65% over 28 days in laboratory and pilot-scale studies, respectively. In landfarming assays, an 85% TPH reduction was observed at the laboratory scale over 6 months, while a 42% reduction was achieved in 3 months during full-scale bioremediation (Frutos et al. 2012). In another study, Brown et al. (2017) applied landfarming technology with nutrient additions for bioremediation of soil contaminated with petroleum hydrocarbons, achieving a notable 53% TPH removal after 16 weeks. In addition, in this approach, which combined oil-degrading microbes, nutrients, and surfactants with subsequent chemical oxidation with 5% KMnO_4_ as a posttreatment, demonstrated successful TPH reduction with an overall efficiency of 92–93% (Bajagain et al. 2020).

Despite its effectiveness in reducing soil pollutants, landfarming faces challenges such as maintaining optimal conditions at larger scales and limitations in treating soils with toxic volatile compounds or mineral pollutants. Drawbacks include the need for large operational areas and decreased microbial activity in adverse conditions. Additionally, it is less efficient in removing soluble pollutants and is particularly unsuitable for treating harmful volatile substances in hot climates. Critics have noted that landfarming can be time-consuming and less efficient compared to some ex situ bioremediation techniques (Azubuike et al. 2016; Saeed et al., 2023).

### Composting

Composting technique is a remediation approach which combines the excavation and blending of contaminated soil with non-hazardous organic materials, such as agricultural waste, animal manure, yard waste, or food processing waste (Gaspar et al. 2012; Jabbar et al. 2022; Raju et al. 2017).

Also, this technique offers a sustainable approach by enabling eco-friendly disposal of organic waste while enhancing the biodegradation rate of contaminants like PAHs. The efficiency of composting depends on factors such as substrate bioavailability, environmental conditions, oxygen presence, and nutrient availability (Sayara and Sánchez 2020).

These amendments, typically at a ratio of 75% contaminated soil to 25% compost, contribute to soil enhancement by reducing bulk density, increasing porosity, facilitating oxygen diffusion, augmenting water holding capacity, and supplying nutrients and organic matter to encourage microbial populations capable of degrading pollutants (Raimondo et al. 2020).

Compost amendments offer quick cleanup, within weeks rather than months. The efficacy of composting in removing total petroleum hydrocarbons (TPHs) from soil has been validated through laboratory and field studies. For instance, studies show a 1:10 mixture of diesel oil–contaminated soil and bio-waste reduced diesel content by 85% after 12 weeks of composting, compared to 35% in soil at ambient temperature (Van Gestel et al. 2003). Composting benefits include nutrient enrichment, improved soil quality, moisture retention, pH adjustment, and microorganism growth. However, it requires extensive monitoring, energy, and a large space and can emit greenhouse gases, with optimal conditions taking 6 months to 2 years (Chukwunonso Ossai et al. 2022).

## Phytoremediation

Phytoremediation is a green and sustainable approach for soil remediation as compared to other conventional soil remediation techniques (Khan et al. 2023; Sharma et al. 2025). Phytoremediation was introduced in 1983 and remains in the testing phase, but it is effective for treating low to moderately metal-contaminated soils (Tonelli et al. 2022). Enhancements like using high-biomass plants with microbes, genetic engineering, and integrating with other remediation methods can improve efficiency (Ashraf et al. 2019; Saxena et al. 2020).

In addition, phytoremediation plays a crucial role in enhancing soil carbon sequestration. Plants used in phytoremediation not only extract and stabilize pollutants but also contribute to the accumulation of organic carbon in the soil through root biomass and the decomposition of plant residues. By promoting healthy vegetation growth in contaminated soils, phytoremediation supports the formation of soil organic matter, which acts as a long-term carbon sink, mitigating greenhouse gas emissions and contributing to climate change adaptation (Adnan et al. 2025).

Phytoremediation also uses plants to remove, stabilize, or detoxify environmental pollutants like heavy metals and organic compounds. Through mechanisms like accumulation, extraction, degradation, stabilization, and evaporation, plants effectively remediate contaminants (Azubuike et al. 2016; Kuiper et al. 2004). This method gained popularity due to its simplicity, cost-effectiveness, and aesthetic appeal. Unlike physical and chemical treatments, phytoremediation improves the physicochemical and biological quality of soils (Ali et al. 2013; Mahmood et al. 2015; Singh and Singh 2017).

Plants and their rhizosphere organisms adapt based on the environmental conditions, pollutant type, and plant capabilities. The two main techniques are phytoextraction, where plants absorb contaminants into shoots and leaves, and phytostabilization, where plant roots immobilize or stabilize contaminants in the soil. By promoting plant health and modifying pollutant access, phytoremediation offers an effective and sustainable approach to managing environmental pollutants, especially heavy metals and organic compounds (Li et al. 2019; Liu and Song 2018; Sánchez-Castro et al. 2023).

Phytoextraction is an effective remediation technique for metal contaminants, where plants absorb pollutants from the soil and accumulate them in their above-ground biomass. Two types of plants suitable for phytoextraction are hyperaccumulators, which produce minimal biomass but concentrate high levels of metals in their tissues, and high biomass plants, which tolerate and accumulate lower metal concentrations but generate more harvestable material (Chandra et al. 2017; Liu et al., 2018; Shiri et al. 2015). Zhuang et al. 2007) evaluated the phytoextraction abilities of six high-biomass plants and metal hyperaccumulators in paddy fields contaminated with Pb, Zn, and Cd. *Viola baoshanensis* accumulated 28 mg kg⁻^1^ of Cd, and *Sedum alfredii* accumulated 6279 mg kg⁻^1^ of Zn, with total extractions of 0.17 kg ha⁻^1^ for Cd and 32.7 kg ha⁻^1^ for Zn. Among high biomass plants, *Rumex crispus* extracted 26.8 kg ha⁻^1^ of Zn and 0.16 kg ha⁻^1^ of Cd, while no plants efficiently phytoextracted Pb (Zhuang et al. 2007).

Phytostabilization uses metal-tolerant plants, known as fixing plants, to stabilize or immobilize heavy metals. These plants remediate the soil through processes such as root uptake, exudation complexation/precipitation, rhizosphere reduction, and soil stabilization (Liu and Song 2018). This method is particularly beneficial in areas with high pollutant concentrations where traditional phytoremediation methods, such as phytoextraction, may not be effective.

Additionally, phytostabilization is often combined with chemical stabilization techniques, incorporating materials such as lime, phosphate, and compost to reduce the bioavailability and toxicity of heavy metals (Bolan et al. 2011). These amendments improve soil pH, enhance nutrient status, and increase water retention, creating a balanced environment for plant growth while mitigating heavy metal pollution. Lee et al. (2014) found that Fe-rich amendments significantly reduced soluble and extractable heavy metals in Pb/Zn mine tailings, with furnace slag and *Miscanthus sinensis* decreasing CaCl₂-extractable metals by 56–91%. Red mud combined with *Pteridium aquilinum* reduced bioaccessible Pb to 34% of its total concentration, while water-soluble Cd, Cu, Pb, and Zn were reduced by 99%. The study indicated that *Miscanthus sinensis* accumulated heavy metals mainly in its roots, making it suitable for aided phytostabilization (Lee et al. 2014).

Choosing appropriate plants is crucial for effective phytoremediation. Ideal plants should have specific traits such as rapid growth, extensive root systems, substantial biomass, tolerance to high metal concentrations, strong metal accumulation capabilities, resilience to extreme salinity and/or pH levels, and significant biomass production rates (Conesa et al. 2007). Therefore, native plant species adapted to metallic conditions are often preferred due to their adaptability to the local environment and favorable growth characteristics (John J. Mellem 2012; Li et al. 2019).

To enhance the effectiveness of phytoremediation and promote the sustainability of the environment, innovation strategies should focus on advancements in molecular biology and genomics, which will enable the development of more efficient, eco-friendly, and long-term solutions for environmental restoration. Developing transgenic plants with superior metal-chelating capabilities and enhanced stress tolerance can significantly improve remediation outcomes. Combining traditional breeding techniques with gene-editing tools like CRISPR (clustered regularly interspaced short palindromic repeats)-Cas9 (CRISPR-associated protein 9) allows for the precise modification of plant traits associated with metal tolerance and accumulation (Barik et al. 2024; Tripathi and Pirzadah 2024).

Additionally, integrating phytoremediation with plant–microbe symbiosis by inoculating plants with metal-tolerant microorganisms and plant growth–promoting rhizobacteria can boost the efficiency of contaminant uptake and detoxification (Sharma et al. 2023). Advanced research into root chemistry and plant–microbe interactions under stress conditions is essential for optimizing metaorganism functionality in contaminated environments. Lastly, creating a database of metal-tolerant genes through genome sequencing and applying this knowledge in phytoextraction technology can clear the way for more effective and commercially viable phytoremediation solutions.

## Biopile Technology

**Fig. 1:**
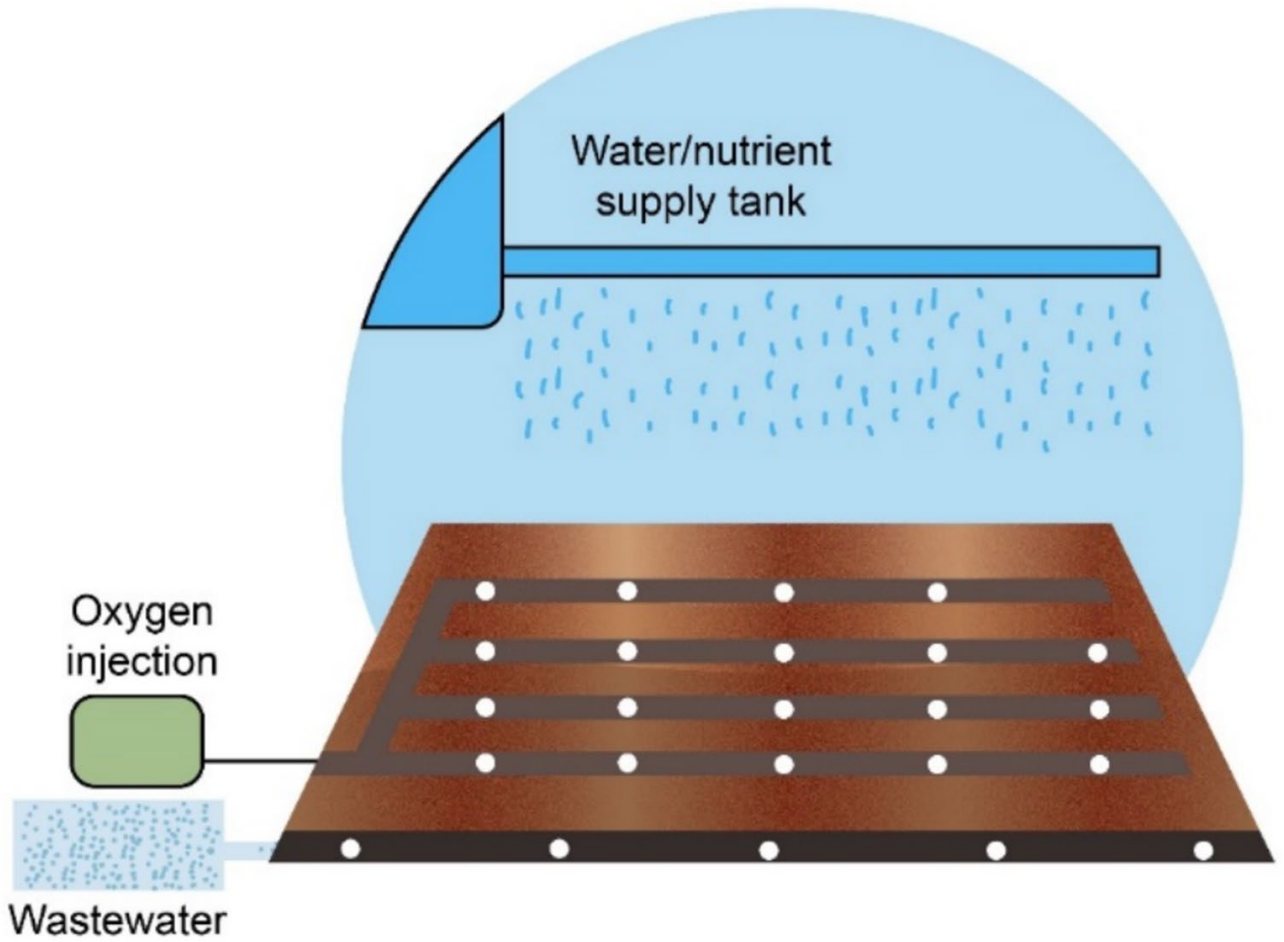
Biopile remediation method for contaminated soil

Biopile is a green and sustainable technology for soil remediation that employs indigenous microorganisms, such as bacteria and fungi, to efficiently decompose pollutants, as shown in Fig. 1 (Zhu et al. 2024). Several organic pollutants, such as petroleum hydrocarbons and motor oil, among others, have been effectively removed by using a biopile (Genovese et al. 2008).

Contaminated soil is dug up and arranged into designated piles, where it is aerated to activate microbial activity for aerobic contaminant degradation. Biopiles are structured to maintain optimal conditions, including temperature, moisture, aeration, and nutrients, to accelerate the biodegradation process. In most cases, native microorganisms carry out the degradation; however, if needed, bioaugmentation can be used to enhance the process.

Biopile, similar to landfarming, is an engineered above-ground system that uses oxygen to stimulate the growth of aerobic bacteria to break down organic components. While landfarming is typically aerated by tillage, biopile aeration is most commonly achieved by forcing air through perforated tubes, although manual aeration can also be applied in small-scale systems (Gutiérrez-Benítez et al. 2023; Jabbar et al. 2022). Biopile has been effective in reducing the concentrations of various petroleum product compounds (Jørgensen et al. 2000), with lighter molecular weight often removed by evaporation, intermediate molecular weight degraded through biodegradation, and heavier molecular weight requiring more time to degrade (Goetz and Brenner 2002). Gallego et al. (2011) presented the effectiveness of the biopile by achieving a reduction in TPH concentration from 5000 to 500 mg/kg in 5 months. Also, Jabbar et al. (2019) achieved a removal efficiency of more than 75% over a 35-day period with a bacterial consortium and nutrient addition.

Liu et al. (2021) assessed biopile processes for weathered diesel oil (WDO)–contaminated soils. The biopile with mixed supplements demonstrated the highest TPH removal efficiency (79%) over 42 days, followed by the red bran addition group with a removal efficiency of 73% over the same number of days. Metagenomics revealed *Sphingomonas* and *Pseudomonas* involvement in degrading heavy components (C_24–30_) and light petroleum hydrocarbon components (C_10–16_), respectively (Liu et al. 2021).

Zhu et al. (2024) demonstrated that biopiling with a mixture of municipal sludge, manure, and agricultural waste achieved high degradation rates of TNT (93.7%), RDX (79.5%), and HMX (92.0%) within 15 days. The research also showed that anaerobic conditions accelerated TNT reduction and led to an increase in genes associated with nitrotoluene reduction, such as *nemA and porA* (Zhu et al. 2024)*.*

Wang et al. (2024) explored the full-scale application of extracellular enzymes from white rot fungi for the bioremediation of PAH-contaminated soil using a hydrogel microenvironment combined with a biopiling system. The remediation targeted 12 PAHs, with benzo[a]pyrene levels exceeding the acceptable limit for construction land. After 7 days, benzo[a]pyrene levels dropped from 1.50 to 0.51 mg kg^−1^, achieving a 66% removal rate and meeting class I screening standards. Microbial analyses revealed enhanced biodiversity, with increased abundance of PAH-degrading bacteria (*Marinobacter*,* Pseudomonas*,* Truepera*) and fungi (*Thielavia*,* Neocosmospora*,* Scedosporium*), providing valuable insights into the practical application of immobilized enzyme and biopiling technology for soil remediation (Wang et al. 2024).

### Key parameters for sustainable biopile and carbon sequestration

The effectiveness of biopile remediation is governed by several key parameters, including soil temperature, pH, soil texture, water content, and aeration. These factors play a critical role in optimizing microbial activity, contaminant degradation rates, and overall system performance. Understanding and controlling these parameters not only enhance the efficiency of pollutant removal but also contribute to broader sustainability goals. By improving soil health and supporting microbial communities, biopile technology can facilitate long-term carbon sequestration through the stabilization of organic matter and the incorporation of carbon-enriched amendments. Moreover, sustainable management of these parameters reduces the environmental footprint of remediation processes to make biopiling a valuable tool for ecological restoration and climate change mitigation.

#### Soil Temperature

The success of biodegradation in the soil biopile technique depends on the optimization of environmental conditions. Bacterial growth is strongly influenced by temperature, with higher temperatures accelerating biodegradation. Temperature also plays a crucial role in hydrocarbon biodegradation, as it affects the chemistry of the contaminant (Chen et al. 2015; Gandhi et al. 2022; Ma et al. 2016).

Optimum degradation rates are usually observed in soil in the temperature range of 10–45 °C (Wu and Coulon 2015). An increase in temperature increases the solubility, decreases the viscosity, and facilitates the mass transfer of hydrocarbons (Aislabie et al. 2006). Optimizing soil temperature also contributes to enhanced carbon sequestration, as healthier microbial communities facilitate the stabilization of organic matter and promote long-term carbon storage in the soil (Singh et al. 2024).

In colder regions, even warming the soil to 5 °C can help biological recovery. Heat is produced as a by-product of the microbial decomposition of natural materials (Gandhi et al. 2022; Iqbal 2003; Ma et al. 2016). Chemical and enzymatic reactions exhibit a proportional increase with rising temperature. For heterotrophic aerobic bacteria, the optimal temperature range is usually between 20 and 35 °C (Iturbe and López 2015). In cold regions, biodegradation efficiency can be increased by taking necessary preparations, such as encapsulating a biopile or using specific bacteria (USEPA 2017, USEPA and UEP^2004^).

#### Water Content

The biodegradation process largely depends on water content, which makes up 80 to 90% of the weight of bacterial cell structures and acts as the primary nutrient (Iturbe and López 2015; Nyer 2000). Water content is critical for microbial growth and the translocation of nutrients and by-products during the biodegradation process in soil (Kästner and Miltner 2016). While soil microorganisms need specific moisture for their growth, excessive moisture can hinder air movement in the soil pores, reducing oxygen availability, which is essential for the metabolic processes of aerobic bacteria (Chaudhary et al. 2020; Paria 2008).

Soil moisture has positive impacts on aerobic bioremediation by enhancing the bioavailability of bioavailability mixtures. Maintaining optimal soil moisture not only accelerates pollutant degradation but also supports the microbial activities that enhance carbon sequestration, as the resulting increase in microbial biomass and organic matter stability helps to store carbon in the soil (Alkorta et al. 2017). The ideal moisture level should be between 30 and 80% of soil water holding capacity or roughly 12 to 30% by weight, ensuring optimal conditions (Gandhi et al. 2022; Larik et al. 2016; Walworth et al. 2007).

It is essential to periodically maintain/monitor moisture in the biopile, as the soil can dry out due to increased evaporation during aeration. In areas with high rainfall or poor drainage, there is a risk of excessive moisture accumulation in the biopile, and this should be considered in the design (USEPA 2017, USEPA and UEP 2004; Wu and Coulon 2015).

#### Soil pH

Another key parameter that influences the performance of enzymes and microorganisms is soil pH (Jabbar et al. 2022). For optimal bacterial growth, the soil pH should ideally fall within the range of 6.5 to 7.5, corresponding to the intracellular pH value, and a neutral value of around 7 (Iturbe and López 2015). This is especially crucial in natural environments where pH typically ranges from 5.0 to 9.0, facilitating the biodegradation of pollutants. Soils with pH values outside this range should be amended by adding lime to increase pH or sulfur to decrease it during biopile operations (USEPA 2017, USEPA and UEP 2004).

#### Soil Aeration

Aeration plays a crucial role, promoting oxygen, moisture, and heat transfer, while microorganisms consume oxygen and water, producing heat. Aeration increases biodegradation, especially for petroleum hydrocarbons, but excessive airflow can lead to heat and moisture loss (Monica et al. 2010).

Behdarvandan et al. (2024) demonstrated that combining aeration and sand addition significantly enhances bioremediation of oil-polluted soils. The results showed an 80% reduction in total petroleum hydrocarbons (TPH) in both fresh and aged polluted soils, compared to a 48% reduction without these treatments. These findings offer valuable insights for designing efficient bioreactors for soil remediation (Behdarvandan et al. 2024).

Soil aeration is crucial and depends on factors such as water drainage, soil respiration rate, soil characteristics, and pH. However, if the ambient temperature is suboptimal for microbial activity, increased airflow may not enhance biodegradation (Dorst et al. 2021). To optimize the restoration system, it is important to understand how increased airflow affects moisture content and internal temperature within the pile. Furthermore, these factors are related to microbial activity, and natural airflow within a pile is mainly driven by wind-induced pressure gradients (Monica et al. 2010).

Biodegradation processes differ based on the presence of oxygen, and some occur under aerobic and anaerobic conditions (Kästner and Miltner 2016). Most microorganisms rely on oxygen as the primary electron acceptor to decompose organic compounds under aerobic conditions. If the oxygen level in the soil drops below 2 mg/L, it creates a favorable environment for anaerobic processes (Iturbe and López 2015). Bacteria rely on a metabolic process to produce energy and require a terminal electron acceptor (TEA) to enzymatically oxidize a carbon source to carbon dioxide (Ladino-Orjuela et al. 2016; Lie et al. 1999). Microbes are classified according to the carbon and TEA sources they use (Purkamo et al. 2017; USEPA 2017, USEPA and UEP 2004). Oxygen generally increases petroleum hydrocarbon metabolism, leading to the degradation of up to 90% of organic compounds under aerobic conditions compared to about 25% under anaerobic conditions. Maintaining sufficient oxygen levels through proper aeration supports rapid microbial activity, which not only improves biodegradation efficiency but also enhances the stabilization of soil organic matter, aiding in carbon sequestration (Abatenh et al. 2017). Availability of oxygen is critical to maintain rapid microbial activity during aerobic hydrocarbon bioremediation (Naseri et al. 2014).

#### Nutrient Content

Microorganisms depend on inorganic nutrients such as nitrogen and phosphorus for growth, enzyme production, and efficient biodegradation, which accelerates pollutant breakdown and supports essential metabolic functions (Albayati and Doyle 2014; Raju et al. 2017; Singh et al. 2012; Wu and Coulon 2015). The solid composition of bacterial cells includes carbon, nitrogen, hydrogen, phosphorus, and smaller amounts of potassium, calcium, magnesium, chloride, iron, and other elements. Carbon, constituting 50% of the bacterial cell, is the main component. For successful degradation, pollutants must contain carbon (Iturbe and López 2015; Suthersan 2001). Nitrogen sources in microorganisms include proteins, cell wall components, and nucleic acids, while phosphorus, in the form of phosphate, is used for synthesizing phospholipids and nucleic acids (Dias et al. 2015).

Nutrient concentrations, including bioavailable carbon, nitrogen, and phosphorus (C:N:P), total organic matter, and oxygen levels, as well as the thermal range experienced by different soils, vary significantly (Ma et al. 2016). Nutrient availability in the soil is influenced by a wide range of biotic and abiotic processes. The optimum ratio of carbon-to-nitrogen-to-phosphorus for biodegradation is usually in the range of 100:10:1 to 100:1:0.5, which varies with the specific compounds and microorganisms involved (Wu and Coulon 2015).

Monitoring the concentration of these nutrients over time allows the observation of microbial activity. NH_4_ can be a source of nitrogen, which undergoes nitrification and volatilization for microorganisms. Additionally, NO_3_^−^ can act as a source of nitrogen, undergo denitrification, and leach from the soil. Organic and inorganic nitrogen sources, such as nitrate salts or urea, are essential in bioremediation as they enhance microbial activity and accelerate contaminant degradation. D’Uva et al. (2021) explored various urea sources, including lab-grade urea, fertilizer urea, and diesel exhaust fluid (DEF), demonstrating that these compounds can serve as nitrogen supplements. Their study highlighted variations in elemental composition among different urea sources, which may influence their effectiveness in remediation strategies (D’Uva et al. 2021).

Microbes effectively use PO_4_^3−^, which is quickly absorbed into the soil. These processes collectively contribute to the dynamic cycle of nutrients in the soil ecosystem. Biostimulation, especially with nitrogen, can increase the effectiveness of bioremediation by reducing the lag phase and maintaining a high microbial population (Raju et al. 2017). Soil amendments such as compost, by-products, biochar, and iron nanoparticles boost hydrocarbon degradation, with nutrients and biochar enhancing hydrocarbon-decomposing bacteria and organic compound biodegradation (Chaudhary et al. 2021). It is important to note that the use of biochar and nutrient-rich amendments can improve carbon sequestration by stabilizing carbon in the soil, thereby enhancing the sustainability of biopile remediation processes (Shar et al. 2025).

#### Microbial Communities

Soil is naturally rich in microorganisms, including bacteria and fungi (Pandey et al. 2018; USEPA 2017, USEPA and UEP 2004). Microbial communities play a vital role in mitigating environmental pollution and manipulating toxic pollutants. The interactions between hydrocarbon-degrading microbial groups and microbial consortia are essential (Speight and El-Gendy 2017). The use of microorganisms in bioremediation has been emphasized due to their role as nature’s main degraders (Fadhile Almansoory et al. 2015). Microorganisms are highly effective in biodegradation due to their diverse species, abundance, and ability to adapt to challenging environmental conditions (Chaudhary and Kim 2019). Their ability to convert synthetic and natural chemicals into energy sources makes them promising in environmental biotechnology (Varjani and Upasani 2017).

Bacteria are the most numerous and play a key role in biochemical processes, even in low-oxygen environments (USEPA 2017, USEPA and UEP 2004). They rely on carbon for growth and an energy source to maintain metabolic functions, requiring nitrogen and phosphorus for cell growth. Microbial communities not only enhance pollutant degradation but also contribute to carbon sequestration by incorporating atmospheric CO₂ into soil organic matter during microbial respiration and biomass synthesis (Mandal et al. 2021).

Genomics and metagenomics are important tools in bioremediation. By using genome information, synthetic bacterial consortia can be designed to improve biodegradation (Lee and Kalia 2020; Macchi et al. 2021). In addition, metabolic network analysis helps to understand the ecological role of each bacterium, and RT-qPCR, together with metaproteomic assays, confirms these roles in the laboratory. Metagenomics also makes it possible to monitor the microbial community during operation and to evaluate the performance of inoculants (Macchi et al. 2021; Pandolfo et al. 2024).

Petroleum hydrocarbon biodegradation follows a complex metabolic pathway, often requiring a microbial community with diverse catabolic abilities. This method has been extensively studied for its effectiveness in degrading petroleum hydrocarbons, especially when a single species cannot effectively break down certain compounds on its own (Chaudhary and Kim 2019). Foght et al. (1990) and Paudyn et al. (2008) studied Prudhoe Bay crude oil and found a 90% degradation of the aliphatic fraction using a two-strain cultivation. Biodegradation efficiency is attributed to the collective action of multiple microorganisms with diverse biodegradation abilities, emphasizing the superiority of a consortium over a single microorganism (Foght et al. 1990; Paudyn et al. 2008).

Xia et al. (2019) observed that a bacterial consortium achieved the maximum crude oil degradation efficiency (85.26%) within 15 days, surpassing single strains such as *Rhodococcus erythropolis* OSDS1 (54.9%), *Alcaligenes *sp. OPKDS2 (63.7%), and *Serratia proteamaculans* S1BD1 (68.0%). In a recent study by Chen et al. (2020), a consortium with two strains achieved more than 95% crude oil degradation in 10 days, demonstrating higher efficiency compared to single strains in breaking down different hydrocarbons.

Raju et al. (2017) conducted pilot-scale experiments using mixed cultures of *Bacillus* strains to remove TPH from soils contaminated with used lubricating oils and crude oil, achieving around 84% and 28% removal after 90 days, respectively. Faggo et al. (2020) tested a mixed culture of *B. subtilis* and *P. aeruginosa* for the biodegradation of light oil at three levels (5%, 10%, and 15%), showing the highest degradation at the 5% concentration with 18 fully degraded compounds from C_10_ to C_25_ at 5% crude oil, while C_8_ to C_11_ and C_8_ to C_9_ were only degraded at 10% and 15%, respectively, over 56 days (Ramadass et al. 2015).

Some studies have suggested the use of specific fungi for the biodegradation of organic pollutants based on laboratory tests (Lin et al. 2010). According to Table 1, various microbial species, including *Pseudomonas *spp.,* Rhodococcus *spp.,* Alcanivorax borkumensis*, and *Mycobacterium *sp., and fungi, such as *Aspergillus niger* and *Penicillium chrysogenum*, have demonstrated the ability to degrade different types of hydrocarbons, underscoring the diverse ecological strategies employed by microorganisms in bioremediation processes and their potential role in enhancing carbon sequestration. Nevertheless, given the large number of microorganisms and studies reported in the literature and, also, the limitations of both the table and the paper, it is not possible to include them all; hence, Table 1 presents a selection of the most representative examples.

**Table 1:** Microbial diversity in organic contaminat biodegradation

Microbial community	Microbes	Type	Role in carbon sequestration	Type of hydrocarbon	References
Hydrocarbon-degrading bacteria	*Pseudomonas aeruginosa*, *Pseudomonas lurida*, *Pseudomonas putida*, and *Rhodococcus erythropolis*	Bacteria	Degrade hydrocarbons, promoting carbon cycling and sequestration	*n*-hexane, *n*-octane, *n*-decane, benzene, toluene, phenol, xylene, PAHs, and diesel	Chen et al. (2020), Gani and Rahman (2024), Lang et al. (2016), Otenio et al. (2005), and Singh and Fulekar (2010)
Oil-degrading bacteria	*Burkholderia cepacia*	Bacteria	Break down oil compounds, enhancing carbon cycling and storage	Trichloroethylene, phenanthrene, and naphthalene	Callicotte (1995), Parales et al. (2000), and Sung et al. (2005)
Polycyclic aromatic hydrocarbon (PAH)–degrading bacteria	*Mycobacterium* sp. and *Rhodococcus* sp.	Bacteria	Facilitate the breakdown of PAHs, indirectly aiding carbon sequestration	*n*-alkanes, PAHs, kerosene, gasoline, and diesel	Brzeszcz et al. (2024) and Van Hong Thi Pham et al. (2018)
Fungal decomposers	*Aspergillus niger* and *Penicillium chrysogenum*	Fungi	Contribute to lignin degradation and plant residue breakdown, enhancing soil organic carbon content	Crude oil, octane, decane, dodecane, ethylbenzene, butylbenzene, naphthalene, acenaphthene, and benzo[a]pyrene	Govarthanan et al. (2017) and Ojewumi et al. (2018)
Thermophilic bacteria	*Thermophiles* (e.g., *Thermus thermophilus*)	Bacteria	Facilitate decomposition at higher temperatures, promoting carbon cycling	Petroleum hydrocarbons, PAHs	Bhattacharya and Gupta (2022)
Soil-borne microorganisms for biodegradation	*Alcanivorax borkumensis* and *Nocardia spp.*	Bacteria	Help in organic carbon mineralization and sequestration	Petroleum hydrocarbons, crude oil, PAHs, and phenol	Azadi and Shojaei (2020), Kadri et al. (2018), and Warr et al. (2018)

#### Pollutant Characteristic

The chemical diversity and complexity of pollutants play a crucial role in determining the rate of biodegradation and, consequently, the efficiency of carbon sequestration (Ren et al. 2018). Pollutants with more complex chemical structures, such as polycyclic aromatic hydrocarbons (PAHs), may degrade at slower rates, limiting the potential for carbon sequestration since slower degradation processes reduce the availability of carbon in the soil (Haritash and Kaushik 2009).

Microbial degradation of organic hydrocarbons varies widely, influenced by their concentration and chemical structure (Martínez-Gómez et al. 2010). High pollutant concentrations can be toxic, inhibiting microorganisms and slowing treatment, while low hydrocarbon levels may limit biodegradation by restricting carbon sources needed for microbial growth (Kavitha et al. 2014; Paria 2008). The susceptibility of these compounds to microbial attack follows a rating: cyclic alkanes < aromatic with low molecular weight < branched alkane < aliphatic alkane (Svinterikos et al. 2019). Studies indicate that lighter hydrocarbon fractions, such as gasoline, break down more readily than heavier oils, such as coal tar or heavier fuel oil, which break down more slowly (Paria 2008; Wang et al. 2019). Essentially, an inverse relationship exists between the complexity of molecular structures and the ease and speed of biological treatment in the technique of biopile for contaminated soils, directly affecting the speed of carbon sequestration (USEPA 2017, USEPA and UEP 2004).

A biopile is considered suitable for treating fuel hydrocarbons and non-halogenated volatile organic compounds. Although halogenated VOCs, semi-volatile organic carbons, and pesticides can also be addressed by biopile, the effectiveness may be different (Gandhi et al. 2022). Soil concentrations exceeding 10,000 to 50,000 ppm for total petroleum hydrocarbons (TPHs) or heavy metals above 2500 ppm are considered inhibitory or toxic to most microorganisms (Omosiowho 2014; USEPA 2017, USEPA and UEP 2004). In cases where contaminant concentrations exceed safe thresholds, simple dilution with clean soil has sometimes been used, but this practice raises sustainability concerns. An alternative approach is the use of bulking agents from renewable organic wastes, such as beef manure, which has been shown to enhance hydrocarbon removal efficiency and reduce degradation time in biopiles (Castro Rodríguez et al. 2022; Fernández et al. 2005). There is a threshold constituent concentration below which bacteria cannot obtain enough carbon to maintain sufficient biological activity. In general, concentrations below 0.1 ppm in soil are considered very difficult to treat by biological treatment alone in a timely and cost-effective manner (Flathman et al. 1994; Pavel and Gavrilescu 2008). Furthermore, achieving a reduction greater than 95% in TPH concentration can be challenging due to the presence of recalcitrant or non-degradable hydrocarbon species (Ball et al. 2012; USEPA 2017, USEPA and UEP 2004).

#### Soil Texture

Soil texture significantly affects the bioremediation process, as it is important to regulate the movement of oxygen, nutrients, and water within the soil, especially in the area where biological activity occurs (Geng et al. 2022; Jørgensen et al. 2000; Salimnezhad et al. 2021). Different soil textures require special considerations to maintain optimal conditions for oxygen addition, nutrient distribution, and moisture within effective ranges (Gandhi et al. 2022). For example, soils with fine particles such as silt and clay tend to clump together, creating challenges in aeration that lead to low oxygen concentrations and problems in the uniform distribution of nutrients, while permeable soils containing sand facilitate faster transport of nutrients, allowing for even distribution of pollutants and nutrients throughout the soil, as well as sufficient space for water and air flow (Davis 2016; Dias et al. 2015).

Porosity, which refers to the amount of open space in soil, extremely affects the efficiency of bioremediation efforts. Increased porosity increases removal efficiency by facilitating better oxygen uptake, air diffusion, and metabolic activity of microbes necessary for pollutant degradation (Wang et al. 2012). Petroleum hydrocarbons tend to strongly adhere to soil particles, especially clays, and their release from soil is considered a limiting factor in their biodegradation process (Jabbar et al. 2022). In soils with high clay content, mixing with a bulking agent such as sand, wood chips, sawdust, or straw may be necessary to improve soil porosity and increase contaminant homogeneity (Davis 2016; Mrayyan and Battikhi 2005; USEPA 2017, USEPA and UEP 2004).

The role of clay in biodegradation is not yet clear. Despite extensive studies on clay’s sorption capacity, which is key to sequestration, few studies directly assess its impact on bioavailability and biodegradation. Some research suggests that the type of clay mineral can influence microbial utilization, with surfactant pre-adsorption onto clays reducing microbial activity in the order of kaolinite < illite < montmorillonite (McAllister and Semple 2010). On the other hand, soils with better aeration, such as sandy soils, allow for greater microbial activity and efficient carbon cycling, enhancing the stability of carbon sequestration (Davis 2016).

Also, the soil surface area (SSA), determined by the type of minerals present, plays a vital role in bioremediation. Soils with higher SSA have greater water holding capacity (WHC), an increased ability to retain pollutants, and a more favorable environment for microbial activity (Khamehchiyan et al. 2007; Macht et al. 2011).

### Advantages and Disadvantages

Biopile bioremediation technology offers several advantages, foremost being its ease of implementation and operation (Galdames et al. 2022). Biopile is particularly effective against persistent environmental contaminants that are difficult to remove or have moderate rates of biodegradation. This technology is suitable for different site conditions and types of pollutants, including oil-based contaminants. Additionally, biopile can also be designed as closed systems, which help control greenhouse gas emissions, aligning with sustainability objectives. Compared to the traditional landfarming method, biopile requires less land area, making it more efficient. In terms of cost, biopile can be competitive with landfill methods and is often preferred due to its ability to remove pollutants rather than trap them. Additionally, biopile technology requires minimal maintenance and monitoring, has low energy consumption, and incurs low costs for municipal services like water and electricity. In ideal conditions, treatment periods vary from 6 months to 2 years, and organic compounds with slower biodegradation rates are effective (Gandhi et al. 2022; Nayak et al. 2022).

While biopile technology offers several advantages, it also has certain disadvantages. Firstly, it requires a significant amount of land for treatment, although it is usually less than the traditional landfarming method. Additionally, volatile compounds may disperse during treatment rather than undergo biodegradation, potentially leading to incomplete remediation. The process also requires excavating contaminated soils, which can be labor-intensive and costly. Biopile may not be effective for remediation sites with high contaminant concentrations, particularly greater than 50,000 ppm of total petroleum hydrocarbons. Moreover, the presence of significant concentrations of heavy metals, usually more than 2500 ppm, may inhibit microbial growth and reduce the effectiveness of bioremediation. In addition, inorganic pollutants and radionuclides are not removed by the biopile technique alone without the combination of other techniques. Furthermore, achieving significant pollutant concentration reductions, such as 95%, and constituent concentration reductions, below 0.1 ppm for soil, can be challenging in this technology (Gandhi et al. 2022; Nayak et al. 2022).

### Biopile combined with other remediation techniques

The integration of biopile technology with complementary remediation methods presents a promising approach to enhancing sustainability and addressing the challenges associated with environmental pollution. Combining biopile technology with other remediation treatments such as phytoremediation or biofilters has demonstrated encouraging results in certain situations (Gandhi et al. 2022; Germaine et al. 2015; Lei et al. 1994). For example, incorporating phytoremediation into the biopile technique can further accelerate the degradation of contaminants such as organic and inorganic compounds through the synergistic interactions between plants and microorganisms (Germaine et al. 2015). Such combinations not only enhance the degradation of pollutants but also contribute to carbon sequestration by promoting microbial and plant-based processes that stabilize organic carbon within soils. Integrating biopiles with biofilters directs volatile hydrocarbon emissions to biofilters, where aerobic microorganisms degrade pollutants, effectively purifying the air (Lei et al. 1994).

In addition, biostimulation, which involves providing additional nutrients or electron acceptors to stimulate microbial activity, may not always yield significant improvements when used alongside biopile technology (Bento et al. 2005; Germaine et al. 2015; Wang et al. 2012). Likewise, bioaugmentation, which introduces specialized microbial cultures to enhance biodegradation, may not achieve the desired outcomes when integrated with biopile technology (Bento et al. 2005; Cunningham and Philp 2000; Wang et al. 2012). In the following, we will examine more of each method in combination with the biopile.

#### Biopile with Phytoremediation

**Fig. 2:**
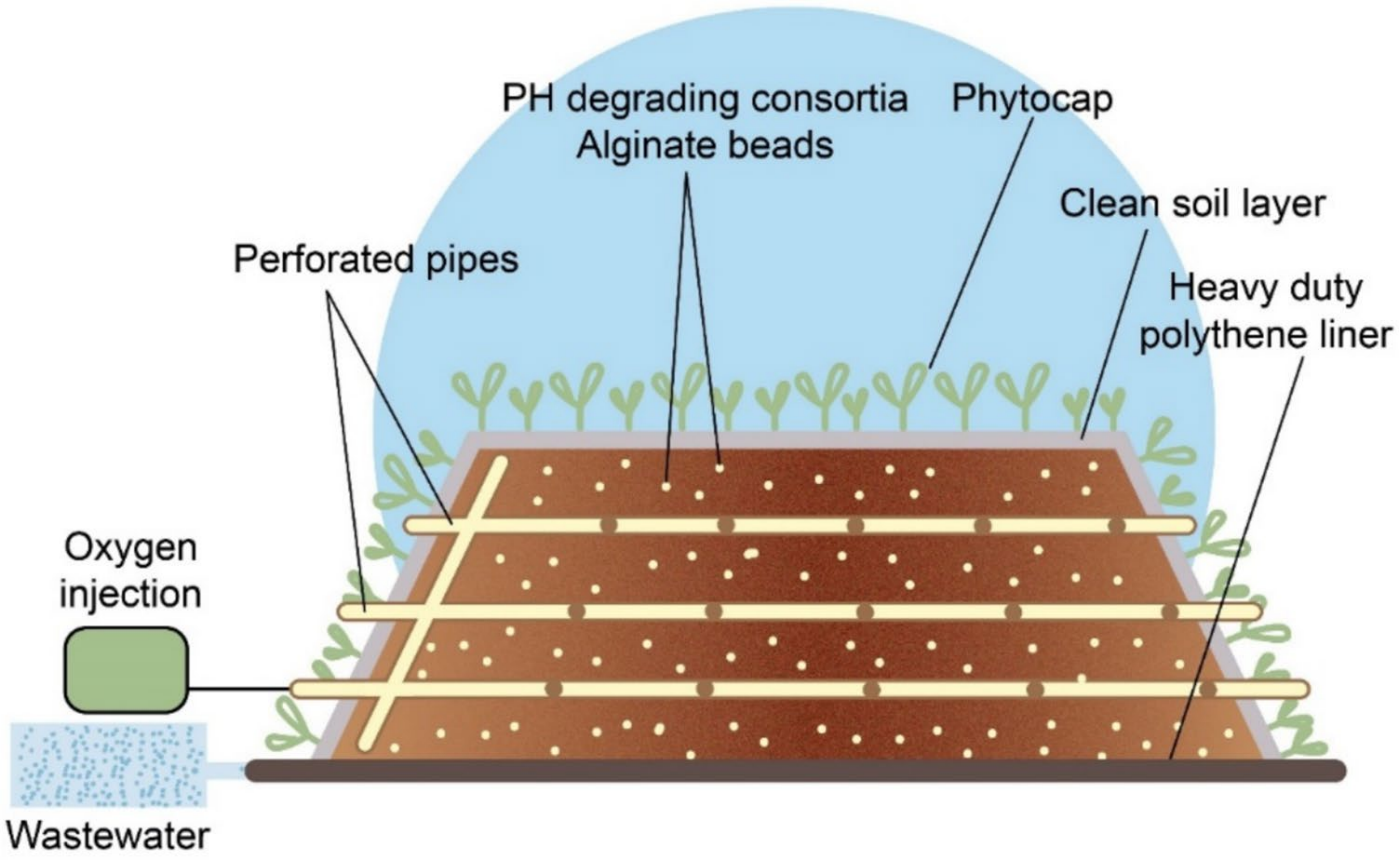
Illustration of the Ecopiles design

The combination of biopile and phytoremediation, known as ecopiling (Conlon et al. 2022; Parmar et al. 2022), has several advantages over traditional remediation methods, as shown in Fig. 2. Ecopiling represents a highly sustainable remediation approach by integrating biostimulation and microbe-assisted phytoremediation to effectively degrade petroleum hydrocarbons and polycyclic aromatic hydrocarbons (PAHs) (Wang et al. 2021). This technique not only enhances the pollutant-degrading potential of microbial communities but also supports plant-based carbon sequestration, contributing to soil health restoration and ecological balance.

Ecopiling is a modification of the traditional passive biopile in that, instead of enclosing the biopile with black plastic, the pile is planted with suitable phytoremediation plants to promote rhizoremediation. In addition, ecopiling promotes rhizophilization, strengthens plant root systems to stimulate microbial activity in the soil, and enhances pollutant decomposition. This multi-dimensional approach targets not only inorganic compounds but also other pollutants such as heavy and toxic metals (Marchand et al. 2016; Martínez-Cuesta et al. 2023). Ecopiling stabilizes the structure of the stand through plant root systems, reducing the risk of erosion and increasing the long-term effectiveness of the reclamation process. It also reduces leachate production through evaporation and transpiration, minimizing the risk of pollutant release (Germaine et al. 2015).

According to Germaine et al. (2015), the ecopiling method, which combines biopiling and phytoremediation, was shown to be highly effective in the remediation of hydrocarbon-contaminated soil. To start with a TPH concentration of 1613 mg/kg in petroleum-impacted soil, the study utilized a biopile amended with nutrients and TPH-degrading bacteria, followed by a phytocap of ryegrass (*Lolium perenne*) and white clover (*Trifolium repens*). After 2 years, TPH levels had fallen to undetectable levels in all but one subsample, which registered a significantly reduced TPH level of 152 mg/kg. These results indicate the substantial potential of this combined approach for large-scale remediation of hydrocarbon-contaminated soils (Germaine et al. 2015).

Martínez-Cuesta et al. (2023) describe ecopiling as a bioremediation approach that combines biopiling with phytoremediation, enhancing hydrocarbon degradation through nitrogen fertilization and bioaugmentation with native bacteria. In their study, ecopiles constructed with hydrocarbon-contaminated soil in Carlow, Ireland, achieved a 99% reduction in aliphatic hydrocarbons and 88% in aromatic hydrocarbons over 18 months. Additionally, microbial diversity, measured by the Shannon index, increased from 7.59 to 9.38, indicating effective biodegradation and adaptation within the microbial community (Martínez-Cuesta et al. 2023).

In 2016, Marchand et al. showed that planting *Medicago sativa* (alfalfa) in ecopiles significantly reduced soil pollutants, including alkanes, PAHs, lead, and copper, compared to unplanted treatments. However, co-planting with *Helianthus annuus* (sunflower) did not increase pollutant removal. Nevertheless, both alfalfa and sunflower had a positive effect on earthworm growth, indicating the effectiveness of ecopiling in remediating soil contaminated with hydrocarbons and trace elements (Marchand et al. 2016).

#### Biopile with Biofilters

**Fig. 3:**
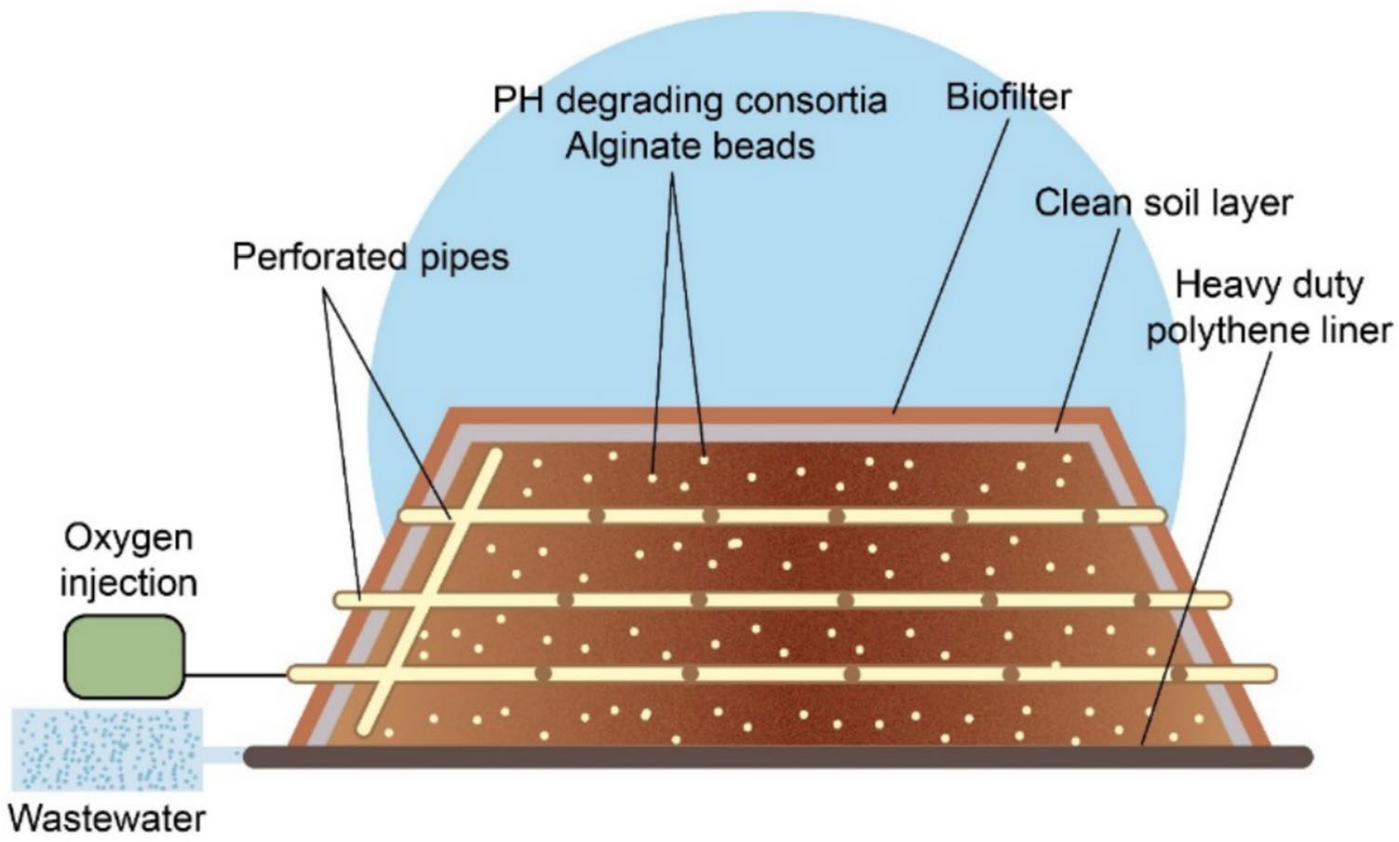
Illustration of the combination of biopile and biofilter methods

Biofilter is a purification technique used to remediate air pollution containing various contaminants such as volatile organic compounds (VOCs), which are released during the bioremediation process (Hazen et al. 2003; Lei et al. 1994; Tyagi and Kumar 2021). In biofilters, pollutants are passed through a media bed containing a variety of microorganisms, such as bacteria and fungi, which play crucial roles in degrading air pollutants. The media bed is typically prepared using materials like bark, composted yard waste, coarse soil, gravel, peat, or plastic shapes, all of which support microbial activity and enhance the degradation of pollutants (Srivastava et al. 2021). The growth and activity of microorganisms in the filter depend on factors such as oxygen levels, absence of toxic compounds, availability of nutrients, humidity, temperature, and pH level (Leson and Winer 1991). Biofilters can combine with biopile because biopile technology may produce pollutant emissions while remediating contaminated soil, as demonstrated in Fig. 3 (Hamby 1996; Lei et al. 1994). Integrating biofilters in biopiles offers a sustainable approach for remediation, ensuring comprehensive cleanup of both soil and air (Srivastava and Kumar 2022). As hydrocarbons in the biopile break down, the released gases are directed to biofilters, where aerobic microorganisms further degrade them before atmospheric release.

Hazen et al. (2003) collaborated with the US Department of Energy and the Institute for Ecology of Industrial Areas of Poland to apply bioremediation to remediate acidic petroleum sludge–contaminated soil at a refinery in southern Poland. They used a biopile system with passive and active aeration, along with nutrient and surfactant application, to enhance biodegradation. A 20–30 cm layer of silty topsoil, planted with grass, acted as a biofilter to capture any VOCs released from the biopile. Over 20 months, more than 81% (120 mt) of petroleum hydrocarbons were biodegraded (Hamby 1996; Hazen et al. 2003; Lei et al. 1994; Sheoran et al. 2022).

### Progress in bioremediation technology

Bioremediation has evolved significantly over the past few decades, with continuous advancements in techniques, materials, and technologies aimed at improving the efficiency and sustainability of pollutant removal from contaminated environments. Initially, bioremediation was limited to basic methods like landfarming and simple microbial degradation; however, with increasing research and technological development, newer approaches have emerged, including bioaugmentation, phytoremediation, and the use of biosurfactants. These technologies have progressively been integrated with each other to form more efficient, sustainable solutions for managing environmental pollutants. Table 2 summarizes the major milestones in bioremediation technology development, explaining key advancements in remediation techniques, materials used, and the environmental impacts of these methods over time.

**Table 2:** Timeline of advancements in bioremediation technology

Year	Bioremediation development	Advancements	Materials/methods used	Environmental impact
1960s	Traditional bioremediation	Early use of microorganisms to degrade pollutants in soils and water	Natural microbial populations	Basic, low-technology approach with limited efficiency
1980s	Landfarming	Landfarming as a widespread method	Organic amendments, including manure and fertilizers	Requires large areas, but simple and inexpensive
1983	Phytoremediation	Use of plants to absorb, degrade, or stabilize contaminants	Plants and natural amendments	Sustainable, low-maintenance method, works well for certain contaminants
1980s–1990s	Biopiles	Biopile and aeration techniques	Air supply, nutrient amendments, and microbial inoculants	Efficient treatment, faster than landfarming, and minimal land use
2000s	Ecopiling & microbe-assisted phytoremediation	Use of microorganisms in conjunction with plant roots	Plants, microbial inoculants, and organic amendments	Increased effectiveness and sustainability of soil remediation
2010 s to present	Integrated bioremediation approaches	Combination of multiple remediation technologies and materials	Nano materials, biosurfactants, biofilters, etc	More sustainable and effective treatment of both air and soil contamination
Ongoing research	Advanced bioremediation	Use of genetically modified organisms (GMOs) and nanomaterials for specific pollutant targeting	GMOs, engineered nanoparticles, and novel bioremediation agents	High precision and efficiency in pollutant removal with reduced environmental impact

## Future directions for sustainable bioremediation

To achieve a sustainable future, bioremediation technologies must evolve to address complex environmental challenges. Emerging techniques such as biostimulation, bioaugmentation, landfarming, and composting remediation are essential to reduce the ecological footprint of remediation efforts. Biostimulation enhances the activity of indigenous microbial communities by optimizing environmental factors and nutrient availability, as demonstrated by recent studies using organic materials like water hyacinth and spent mushroom compost to accelerate petroleum hydrocarbon degradation. Similarly, bioaugmentation introduces specialized microorganisms to contaminated soils, leveraging their enhanced degradation capabilities for persistent organic pollutants and heavy metals, as seen in the successful use of *Arthrobacter aurescens* to remove terbuthylazine from soils. Combining techniques like biopiling and composting with sustainable materials, such as biosurfactants derived from agricultural by-products, can significantly enhance both the environmental sustainability and the effectiveness of these approaches. This integration not only promotes efficient contaminant degradation but also enriches the soil, making it a holistic and eco-friendly remediation strategy.

## Bioremediation and Sustainability: a bibliometric map

Integrating artificial intelligence (AI) techniques into bibliographic analysis to gain new insights into technologies and combined methods for soil remediation significantly increases the scope for optimizing remediation strategies (Ashkanani et al. 2024; Gautam et al. 2023). Without AI tools, researchers have to manually sort through large numbers of studies, a time-consuming and error-prone process, as most of the articles they review turn out to be irrelevant.

To make systematic reviews more efficient, we conducted a literature analysis on soil remediation, focusing on sustainable bioremediation methods, supported by bibliographic analysis to improve prediction accuracy. Based on our evaluation of AI tools, we identified VOSviewer as an effective platform for our research needs. This tool excels in text mining and provides powerful features for visualizing datasets of articles, thus facilitating comprehensive literature analysis. Our aim is to demonstrate the efficacy of novel materials and technologies in the field of bioremediation in recent years, with an emphasis on sustainability. We conducted an analysis of published documents in this field using VOSviewer. This tool analyzes data from multiple article providers, such as Web of Science and Scopus, supporting us in identifying a broad set of relevant articles for our review.

The data analysis was conducted and evaluated based on two principles. The first principle focused on assessing how bioremediation methods can be integrated with other techniques and their effectiveness in the remediation process. The second principle examined how bioremediation methods can contribute to environmental sustainability.

## Sustainability Principle

For the bibliometric analysis of bioremediation, data were extracted from the Scopus database covering the period from 2015 to the present. The search was conducted by using the keywords “soil,” “bioremediation,” and “sustainability,” which were selected to evaluate the role of bioremediation methods in environmentally sustainable practices. This search identified a total of 1128 relevant papers. The dataset was then analyzed by the VOSviewer tool that allows visualization of bibliometric networks and keyword relationships. A minimum threshold of ten keyword occurrences out of 8771 keywords was applied, and this filtering process resulted in 349 relevant keywords for further analysis.

**Fig. 4:**
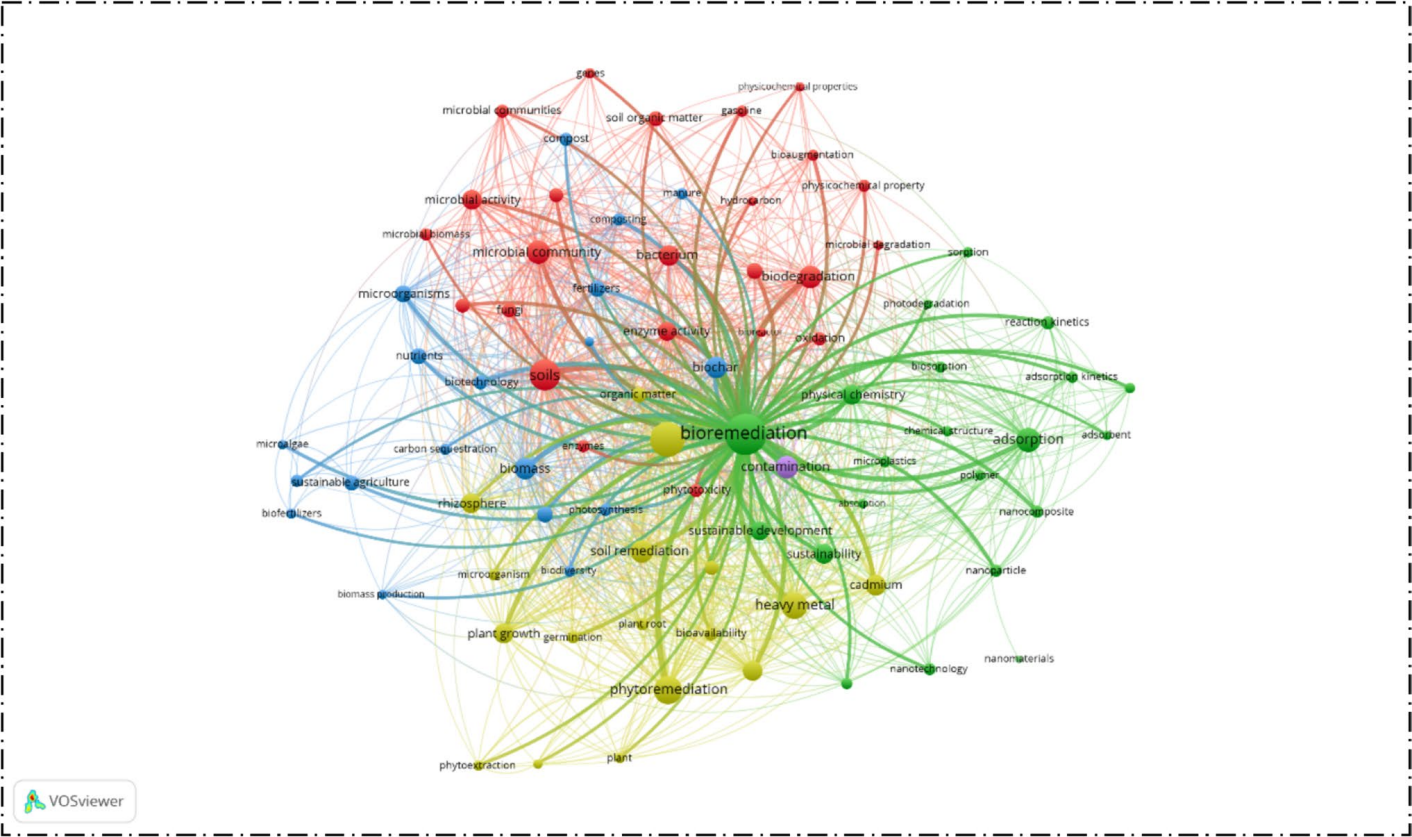
Visualization of bioremediation research trend with a focus on sustainability (2015–present) based on Scopus

The generated keyword co-occurrence maps and citation networks (Fig. 4) provide insights into the major research directions in this field. The graph shows the increasing focus on sustainable and innovative materials such as nanoparticles, biosurfactants, fungi, biochar, and biosorbents that are frequently discussed in relation to bioremediation efficiency and carbon reduction. These materials are recognized not only for their remediation potential but also for their ability to minimize secondary environmental impacts.

This analysis also shows a clear shift in methodological approaches over the past decade. Most recent studies tend to employ combined strategies, especially the integration of biological techniques with physicochemical methods, in order to improve efficiency and adaptability under different site conditions. By contrast, physicochemical methods are reported less frequently because of concerns related to energy consumption, secondary pollution, and sustainability. This trend indicates that the research community is prioritizing bio-based and hybrid solutions as more environmentally responsible options for future remediation practices.

To explore research trends in biopile technology, data was collected from the Scopus database from its inception to the present publications. The search was performed by the keywords “soil,” “bioremediation,” and “sustainability” that were selected to find how biopile technology is studied in relation to environmental sustainability. This search identified a total of 1201 relevant publications. The dataset was analyzed using the VOSviewer tool that provides a map and visualizes relationships among keywords and citation patterns. To refine the results, a threshold was applied so that only keywords appearing at least five times were considered. Out of 1619 total keywords, 104 significant terms met this criterion and were included in this analysis.

**Fig. 5:**
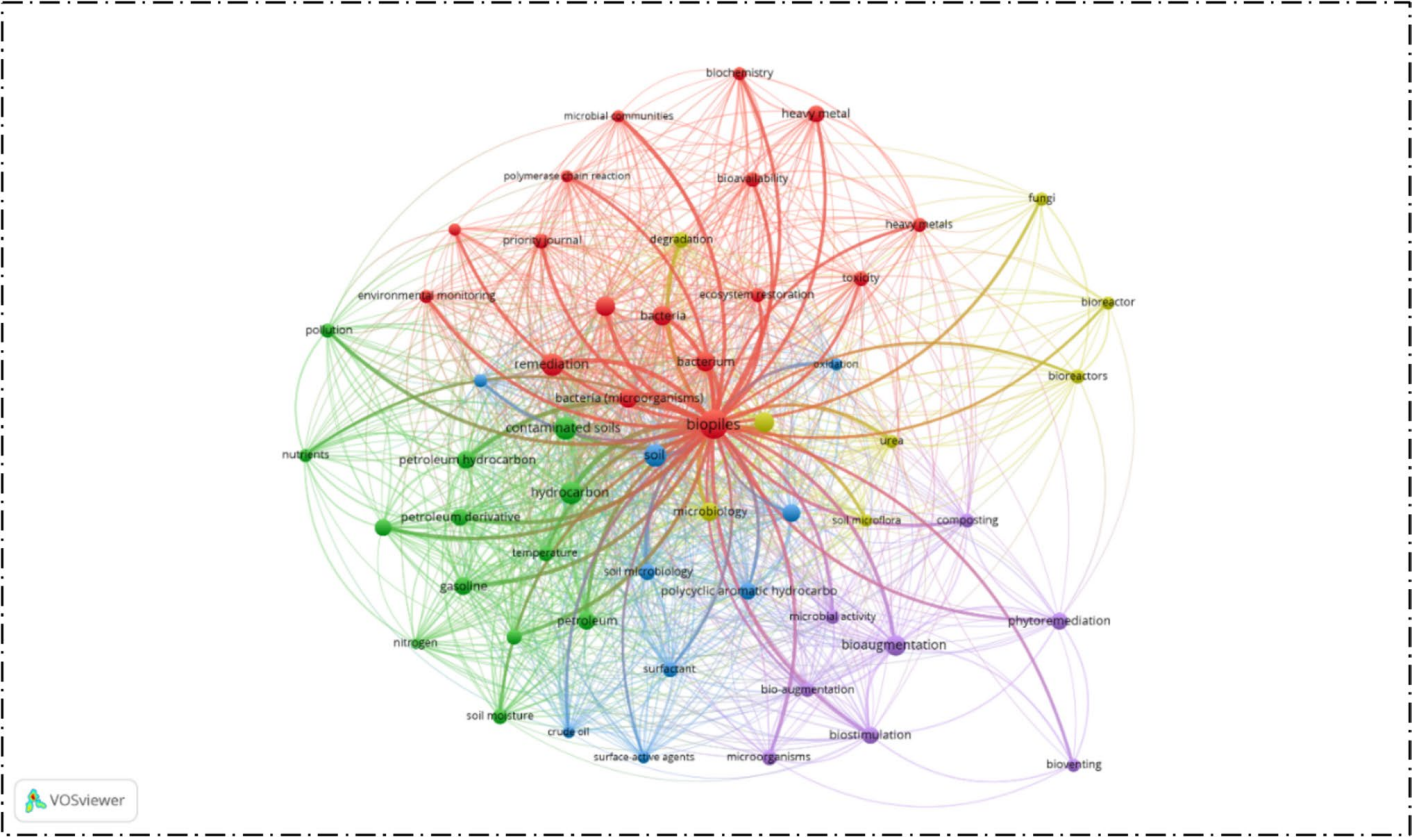
Visualization of biopile research trend with a focus on sustainability based on Scopus

The result of this analysis is presented in Fig. 5. These visualizations reveal that biopile research increasingly focuses on sustainable materials with fungi, surfactants, and biochar appearing as prominent terms. These materials are recognized for their role in improving biodegradation efficiency while maintaining environmental safety. Another clear trend is the growing use of gene-based strategies. Genomics and related approaches are now being applied to better understand microbial communities in biopiles and to improve their performance in breaking down contaminants. Alongside this, the analysis confirms that bioaugmentation and biostimulation have become routine practices in biopile technology. These methods are increasingly combined with other approaches to maximize efficiency and ensure sustainability in soil remediation.

Beyond efficiency in contaminant removal and potential contributions to carbon sequestration, the long-term fate of remediated soils remains a critical issue. Residual toxicity and genotoxicity can persist even after significant pollutant degradation, raising concerns about the safe reuse of treated soils. These risks show the need for further research into reliable monitoring tools and post-remediation assessments to ensure environmental and human safety. In this regard, our bibliometric analysis also reflects the growing attention to these aspects, as terms related to toxicity and genotoxicity emerge within the research network. Addressing these challenges through integrated approaches will be essential for positioning biopile technology as a fully sustainable remediation strategy.

## Combination of Remediation Techniques

The selection of remediation techniques for contaminated soils depends on various factors such as site geography, contamination characteristics, remediation goals, affordability, budget constraints, implementation readiness, time required, and public acceptance (Liu et al., 2018; Ostovar et al. 2023; Saberi et al. 2018). As discussed in previous sections, different soil remediation techniques offer advantages and disadvantages. For example, chemical fixation can be effective in reducing the bioavailability and toxicity of high-concentration heavy metals in heavily polluted sites and allowing plant growth. However, it may not fully restore ecosystem functions and could have harmful effects on the environment. On the other hand, phytoremediation gradually restores ecosystem function by using plants to absorb and accumulate heavy metals, but it may be slower and less effective in highly polluted areas.

Integrated approaches, combining two or more remediation techniques, may be necessary for comprehensive soil remediation. As discussed in the previous section, the combination of biopile and phytoremediation methods can be used to remediate heavy metals from contaminated soils with mixed pollutants. Although the implementation of a biopile is only capable of cleaning organic pollutants, this integrated approach allows for the creation of a more appropriate and effective strategy based on the specific needs of the site. In Table 3, the combination of different remediation methods with the biopile method is discussed. Based on the data of each method and the advantages and disadvantages of each method, it may be inferred that if these methods are combined with the biopile, the advantages and disadvantages for cleaning, productivity, and pollutant control can be evaluated.

**Table 3:** Summary of the advantages and disadvantages of the combination biopile with other techniques

Strategy	Location	Methods	Pollutants	Advantage	Disadvantage
Physicochemical	In situ	Surface capping	Inorganic and organic substances	Reduced long-term maintenance/enhanced remediation effectiveness	Implementation complexity/economic costs
		Encapsulation	Inorganic and organic substances	Long-term effectiveness/enhanced remediation effectiveness	Implementation complexity/economic costs
		Stabilization/solidification	Inorganic and organic substances	Synergistic effects/long-term effectiveness	Complexity and cost/compatibility concerns/implementation challenges
		Soil flushing	Organic compounds/soluble inorganic substances	Enhanced contaminant removal	Potential for off-site impacts/complexity and economic costs/compatibility concerns
		Electrokinetic	Organic compounds/soluble inorganic substances	Enhanced contaminant removal/synergistic effects	Energy consumption/implementation complexity/economic costs
		Chemical oxidation/AOPs	Inorganic and organic substances	Enhanced contaminant removal/synergistic effects/cost-effectiveness	Risks for human health and the environment/complexity
		Thermal	Organic substances	Enhanced remediation effectiveness/time effective/synergistic effects	Energy consumption/implementation complexity/economic costs/increase in contaminants volatilizing
		Chemical fixation	Inorganic and organic substances	Synergistic effects/increased contaminants removal/permanent containment	Compatibility concerns/complexity and economic costs
	Ex situ	Soil washing	Organic compounds/soluble inorganic substances	Enhanced contaminant removal	Waste management/complexity and economic costs
		Landfilling	Inorganic and organic substances	Permanent containment	Time-consuming/economic costs/long-term maintenance/waste volume
Biological	In situ	Natural attenuation	Organic substances	Cost-effectiveness/low environmental impact/adaptability	Time-consuming/limited predictability
		Biostimulation	Organic substances	Enhanced biodegradation/cost-effectiveness/low environmental impact	Time-consuming/requires site-specific optimization
		Bioaugmentation	Organic substances	Enhanced biodegradation/targeted treatment of specific contaminants/adaptability/low environmental impact	economic costs/site-specific optimization/risk of failure
		Bioventing	Volatile organic compounds (VOCs) or simply	Enhanced biodegradation/cost-effectiveness/low environmental impact	Time-consuming/limited applicability to certain contaminants/potential for off-site impacts
		Biosparging	Organic substances	Enhanced biodegradation	Implementation complexity/economic costs/limited applicability to certain contaminants
		Vermiremediation	Heavy metals and organic substances	Enhanced biodegradation/cost-effectiveness/environmental sustainability/improved soil structure	Complexity/monitoring requirements/longer duration/
		Phytoremediation	Inorganic and organic substances	Enhanced contaminant removal/complementary mechanisms/soil stabilization and erosion control/reduced environmental impact	Time-consuming process/maintenance requirements
	Ex situ	Landfarming	Organic substances	Enhanced biodegradation/increased treatment capacity/cost-effectiveness	Slow remediation rates/limited effectiveness for certain contaminants
		Composting	Organic substances	Enhanced degradation/improved soil quality	Slow remediation rates/limited effectiveness for certain contaminants
		Bioreactors	Organic substances	Enhanced contaminant degradation/improved treatment efficiency	Higher cost/complexity/energy consumption
		Windrows	Organic substances	Enhanced degradation/improved oxygen supply/reduced space	Temperature control/limited effectiveness for certain contaminants

In the future, advancements in soil remediation techniques are poised to innovate environmental cleanup efforts. Recently, researchers have increasingly focused on integrating various remediation methods to enhance efficiency and optimize modification processes, as demonstrated in the previous section using artificial intelligence tools. These approaches involve combining traditional techniques such as soil excavation and chemical treatments with innovative technologies like phytoremediation and nanotechnology. By employing the synergistic effects of these various methods, future soil remediation efforts hold the promise of achieving greater levels of pollution control and efficiency than ever before. Additionally, emerging technologies such as bioremediation using genetically engineered microorganisms and advanced monitoring systems powered by artificial intelligence are anticipated to play key roles in optimizing remediation strategies and ensuring long-term environmental sustainability. As interdisciplinary collaboration and technological innovation continue to push forward, the future of soil remediation appears increasingly promising, offering hope for restoring contaminated ecosystems and protecting human health.

## Conclusion

This comprehensive review of soil remediation methods underscores the importance of sustainability in bioremediation. It reveals that the use of sustainable materials, such as nanotechnology, offers promising advancements in soil decontamination. Nanoparticles, biosurfactants, and other innovative materials enhance the efficiency of bioremediation processes, providing more effective removal of pollutants, especially heavy metals and organic compounds. Further research into the application of these sustainable materials will help optimize their use in soil remediation and contribute to the development of eco-friendly technologies.

Sustainable remediation approaches, such as phytoremediation, also play a key role in advancing bioremediation practices. The integration of plants with microbial processes allows for the natural degradation of contaminants while promoting environmental sustainability. Phytoremediation offers a low-cost, energy-efficient solution for addressing pollutants like heavy metals and petroleum hydrocarbons. Future studies should focus on improving plant–microbe interactions and enhancing plant tolerance to pollutants to further increase the effectiveness of phytoremediation in various soil types and environmental conditions.

Finally, combining bioremediation with other remediation technologies can lead to even greater environmental sustainability and remediation success. Synergizing bioremediation with physicochemical methods, such as thermo-chemical treatments, or incorporating biopile technology with phytoremediation, offers a holistic approach to soil cleanup. These combined approaches allow for a more comprehensive treatment of a wider range of contaminants, promoting both efficiency and sustainability. However, more research is needed to fully understand the synergistic effects of these combined methods and to determine the most cost-effective and site-specific solutions for long-term environmental sustainability.

## Supplementary Information

Below is the link to the electronic supplementary material.

ESM 1 (1.10 MB DOCX)

## Data Availability

All data used in this study are available in publicly accessible sources, as referenced in the manuscript.
