# Supplementary material for: Advancements in biopile-based sustainable soil remediation: a decade of improvements, integrating bioremediation technologies and AI-based innovative tools
Source: Environ Sci Pollut Res Int. 2025 Oct 8;32(40):22766–93. doi: 10.1007/s11356-025-37002-1 (PMC12553613; doi:10.1007/s11356-025-37002-1)
Supplement: Supplementary file 1 — (1.10 MB DOCX) [file 11356_2025_37002_MOESM1_ESM.docx]

**Supplementary Section:**

**Advancements in Biopile-Based Sustainable Soil Remediation: A Decade of Improvements, Integrating Bioremediation Technologies and AI-based Innovative Tools**

Mojtaba Ostovar^1^, Sara Muñana^1^, Alazne Galdames^1^, Josu Berganza^2^, Maider Orueta^3^, José Julián Esteban^4^, Pilar Brettes^2^, José Luis Vilas Vilela^1,5^, Leire Ruiz Rubio^1,5*^

^1^Macromolecular Chemistry Group (LQM), Physical Chemistry Department, Faculty of Science and Technology, University of the Basque Country (UPV/EHU), 48940 Leioa, Spain

^2^Gaiker, GAIKER Technology Centre, Basque Research and Technology Alliance, Zamudio 48170, Spain

^3^Iragaz Watin S.A., 20720 Azkoitia, Spain

^4^Departamento de Geología, Facultad de Ciencia y Tecnología. Universidad del País Vasco-Euskal Herriko Unibertsitatea (UPV/EHU), Barrio Sarriena s/n, 48940 Leioa, Spain

^5^BCMaterials, Basque Center for Materials, Applications and Nanostructures, UPV/EHU Science Park, 48940 Leioa, Spain

**Bibliometric and Literature Analysis**

The bibliometric analysis was also conducted using the Web of Science (WoS) database for the period 2015 to the present, applying the same set of keywords (soil, bioremediation, and sustainability). This search identified a total of 487 relevant documents, which together provide a comprehensive overview of global research activities linking bioremediation with sustainability. The dataset was analyzed using the VOSviewer tool to generate keyword co-occurrence and co-citation maps. A minimum threshold of 10 occurrences out of 2,908 keywords was applied, resulting in 70 keywords meeting the criteria. This filtering allowed the identification of dominant themes and materials being explored within the context of bioremediation. The resulting map shows clear patterns that are consistent with the Scopus analysis. Figure S1 present the co-occurrence networks that show clusters of keywords around sustainable bioremediation practices. Prominent materials include nanoparticles, biosurfactants, fungi, biochar, and biosorbents, all of that are repeatedly associated with enhanced degradation efficiency, carbon reduction, and environmentally friendly approaches. These materials appear as core nodes in the network, reflecting their central role in current research. The analysis also reveals temporal trends over the last decade. There is a growing emphasis on combined methods where biological processes such as microbial degradation or fungal bioremediation are integrated with physico-chemical approaches. This combination is seen as a way to increase pollutant removal efficiency while still addressing environmental sustainability. On the other hand, physico-chemical methods (e.g., chemical oxidation or thermal treatment) appear less frequently due to limitations related to cost, energy requirements and the potential for secondary environmental impacts.


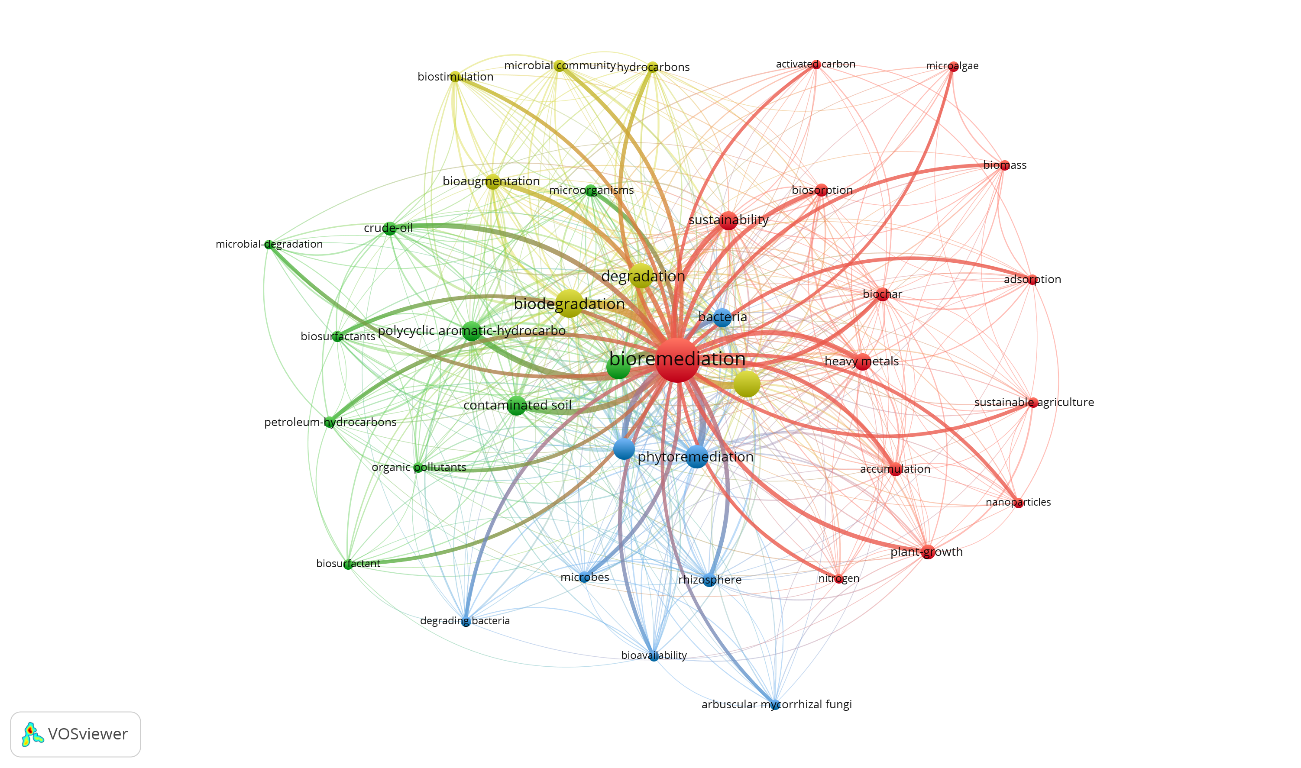
**Fig.S1.** Visualization of Bioremediation Research Trent with a Focus on Sustainability (2015–Present) Based on Web of Science

The bibliometric analysis of biopile technology was also performed by the Web of Science (WoS) database from its earliest records up to the present. The same keywords “soil”, “bioremediation” and “sustainability” were used in the search to ensure comparability with the Scopus dataset. This search identified a total of 228 relevant publications. The dataset was processed by the VOSviewer tool that was applied to examine keyword co-occurrence and citation networks. A threshold was set so that only keywords with at least ten occurrences were included in the analysis. Out of 2,908 keywords, 142 terms met this criterion and were retained for visualization.

The result, illustrated in Figures S2 provides a detailed picture of the main themes in biopile-related research. Similar to the Scopus findings that the analysis shows the central role of sustainable materials such as fungi, surfactants, and biochar. These materials are frequently mentioned in connection with improving the efficiency and environmental compatibility of biopile systems. The Web of Science analysis also reveals an increasing emphasis on gene-based strategies, reflecting the integration of genomics and related approaches into the design and monitoring of biopiles. These strategies are used both to optimize microbial consortia for enhanced degradation and to track the functional performance of biopiles during operation. In addition, the result confirms that bioaugmentation and biostimulation are widely recognized as effective biological interventions as also observed in the Scopus analysis and they are increasingly combined with other methods to enhance remediation outcomes.


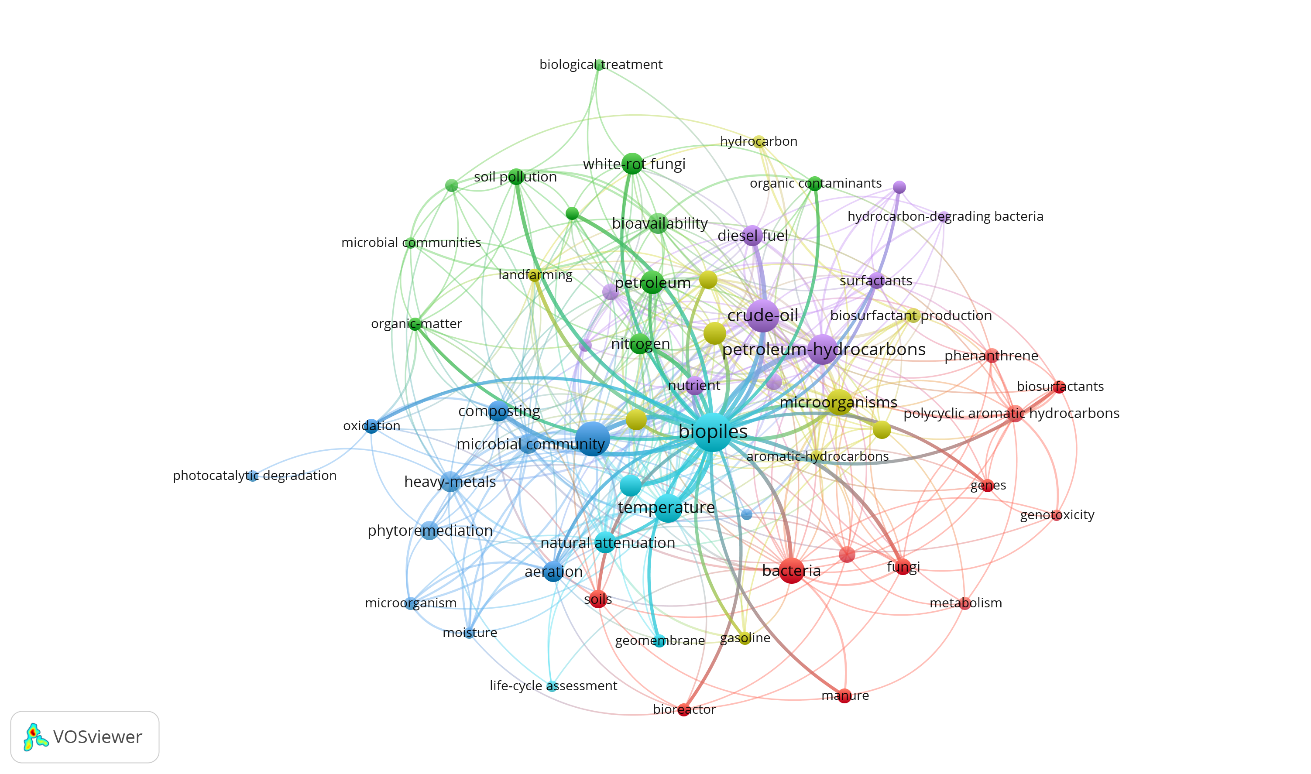
**Fig.S2.** Visualization of Biopile Research Trent with a Focus on Sustainability Based on Web of Science
